# The relationship between stress, trait emotional intelligence and mental health amongst Gaza’s basic-year medical students during the COVID-19 outbreak

**DOI:** 10.1186/s43045-021-00146-0

**Published:** 2021-10-04

**Authors:** Basel El-Khodary, Siham Alshawamreh, Mariam Abu Salah, Amro Hamada, Baraa Alhendawi, Mohammed Alhabil, Younis Alemour, Hadil Zaqout, Ezz Aldeen Wadi

**Affiliations:** 1 Department of Psychology, Islamic University of Gaza, Gaza, Palestine; 2 Faculty of Medicine, Al-Quds University - Al-Azhar branch, Gaza, Palestine

**Keywords:** Stress, Depression, Trait emotional intelligence, Palestinians, Medical students

## Abstract

**Background:**

This study investigates the relationship between exposure to stress, trait emotional intelligence (trait EI) and mental health problems (anxiety and depression symptoms) amongst basic-year medical students during COVID-19. The sample consists of 379 basic-year medical students. Off them, 160 (42.4%) were male and 218 (57.5%) were female. The following measures were used in the study: *The Medical Student Stressor Questionnaire* (*MSSQ*), *Trait Emotional Intelligence Questionnaire—Short Form* (*TEIQue-ASF*), *The anxiety symptoms scale and the depression scale.*

**Results:**

The majority of students reported high to severe level ARS and mild to moderate DRS. Female students showed higher levels of ARS, TLRS, SRS, GARS and anxiety symptoms compared to male ones. Moreover, students with higher levels of academic performance reported lower levels of ARS and DRS, higher levels of trait EI and lower levels of anxiety and depression symptoms. In addition, trait EI has a significant negative association with anxiety and depression symptoms and stress domains (ARS, IRS, TLRS, SRS, DRS, GARS) and total stress. Finally, stress domains are positively correlated with anxiety and depression symptoms.

**Conclusion:**

Basic-year medical students in the Gaza Strip are exposed to stressful events which aggravate the effect of exposure and cause higher levels of anxiety and depression symptoms.

## Background

Stress is, generally speaking, a condition where the demands on an individual exceed their capacity to respond, potentially having negative physical and psychological consequences [1]. Stress can be something positive, up to a certain level, but if stress exceeds that level, it transforms into something negative which affects the health and mental condition by means of an exaggerated reaction of the organs, disturbed adjustment and other health problems [2]. Stress can also lead to social problems such as poor relationships with peers and family members, and cause cognitive problems or deterioration of academic performance [3].

Stress is often seen as a mental process, but it also affects the body physically. According to Hans Selye’s theory, it helps people to be more aware of the physical symptoms of stress if they understand the stages the body undergoes when exposed to stress. General adaptation syndrome (GAS) is a three-stage process that explains the physiological changes the body undergoes under stress: (a) the alarm reaction stage which comprises an initial shock phase and a subsequent counter-shock phase; (b) the resistance stage where symptoms of the alarm reaction disappear indicating the adaptation of the organism to the stressor; and (c) the exhaustion stage which occurs if the stressor persists and the symptoms of the first stage reappear but it is no longer possible to resist [4].

Chronic stress may lead to increased heart rate during sleep [5], diseases such as hypertension, diabetes mellitus, ageing and immunosuppression effects and poor social relationships [3].

There are three stressor categories: (1) frustration, which is felt when the efforts to achieve a goal are hindered by external or internal obstacles or the impossibility of achieving it; (2) conflict, in situations where two opposite motivations or needs occur simultaneously and the achievement of one blocks the achievement of the other and (3) pressure, when the tensions determined by an inner or outer force to increase the pace or effort made to achieve a certain goal or change the strategy needed to achieve it [2].

Medical schools aim to produce competent, well-trained, qualified doctors to help and support patients at all levels, in order to advance medical knowledge and promote public health [6]. However, medical students may be exposed to stressful events during their studies. The prevalence of stress has been reported at 65% amongst medical students in the United Arab Emirates University, 30.9% amongst Egyptian medical students, 57% amongst medical students in King Saud University, Saudi Arabia [7] and 41.9% amongst medical students in Malaysia [8]. Stress, burnout and academic efficacy all play roles in the physical and psychological well-being of preclinical medical students [9]. For example, they are at great risk of depression and anxiety [7, 10]. Despair is a normal physiological effect, which can be experienced after an emotional, pathological or even physical problem. However, if it continues for a long time and affects the function of a person, it can develop into a mental disorder, specifically depression, which is a major cause of death worldwide [11]. Depression has an adverse impact on young people’s lives, causing poor academic performance, substance abuse and suicidal ideation [1].

Meta-analysis shows that the prevalence of psychological stress amongst medical students to be 41.9%, and that it is significantly associated with depression [8]. Recent research in Saudi Arabia indicates a statistically significant positive correlation between high levels of stress and burnout in students with a lower grade point average who are not involved in extracurricular activities [12]. Several systematic reviews suggest a high prevalence of depression and anxiety amongst medical students with overall levels of psychological distress consistently higher than the general population and age-matched peers [13].

Meta-analysis shows that about one in three medical students globally have anxiety symptoms. Administrators and leaders of medical schools should take the lead in destigmatising mental illness and promoting help-seeking behaviours for students who are stressed or anxious [14].

Several studies find a high prevalence of depression and anxiety amongst medical students in Palestine. Depression and anxiety symptoms are shown to be prevalent amongst medical students in a major university in the West Bank of Palestine [15]. Moreover, stress during the first year of medical school has a higher impact than during the following years, especially before and during exams, mainly due to the highly competitive environment, absence of extracurricular activities, absence of a learning strategy, sleepless nights before exams and unhealthy food intake during exams [2]. Previous research shows that academic stage is a predictor of anxiety symptoms amongst students in the basic academic years (years 1-3) [15].

Recent findings suggest that medical education might add to the already stressful lifestyle of university students, negatively affecting their mental health. In the Gaza Strip, where living conditions are extremely harsh, there is concern regarding the mental health of Palestinian students enrolled in both of the only two medical schools available [16].

### Trait emotional intelligence

Trait EI is defined as “the constellation of emotional self-perceptions located at the lower levels of personality hierarchies” [17]. For students, variations in levels of trait EI lead to variations in academic performance and creativity in subject domains [18], in a positive manner [19]. There is also a relationship between trait EI and aggressive behaviour in students, meaning trait EI is an important negative offensive behaviour indicator [20].

Away from the academic field, trait EI has great importance in routine daily life. Students with higher trait EI scores are less likely to experience emotional and behavioural problems [21] and engage in antisocial action [22]. A negative association has been identified between trait El and sensation seeking in teens [23], and it plays a significant role in peer interaction and socio-emotional maturity [24]. Trait EI has a significant positive importance in satisfaction with relationships [25].

### COVID-19 in the Gaza Strip

Unlike many other parts of the Middle East, cases of COVID-19 were not detected until 22 March 2020 in the Gaza Strip. This delay was most likely related to the restricted movement of Palestinians to and from Gaza, due to the blockade imposed on residents, with only two crossing points, in the north to Israel (Eretz) and in the south to Egypt (Rafah), with severe movement constraints at both crossing points [26].

In March 2020, the authorities imposed mandatory isolation for 21 days at designated quarantine centres for those entering by way of Israel and Egypt. Universities in Gaza moved to online-only teaching via Google Meet and Moodle for the full academic year and autumn term, holding the end of year exams online. However, students without access to a computer, laptop or broadband connection were given the chance to undertake their assessments in person.

The obstacles to e-learning in Gaza include weak internet networks, power outages and insufficient awareness amongst students and their families of the importance of e-learning. There is low accessibility to online material and computers or smartphones for some students [27].

### Context of the study

The Gaza Strip is one of the most densely populated areas in the world (1.91 million people live in 365 km^2^) [28]. Up to 4 June 2021, there were a total of 110,479 positive COVID-19 cases in the Gaza Strip, of which 105,368 recovered and 1028 passed away [29].

Basic-year medical students may be exposed to stressful events such as the experience of a new academic life, the obstacles of e-learning, new societal habits, settling in a war zone and socioeconomic problems, all of which put them under great pressure and stress. The aim of the current study is to investigate the relationships between stressful events, trait EI and mental health (depression and anxiety symptoms) amongst basic-year medical students during the COVID-19 pandemic in the Gaza Strip. To the best of our knowledge, this is the first study to examine the relationships amongst these variables during the COVID-19 pandemic.

## Methods

### Participants and procedures

The sample consisted of 398 basic-year medical students (201 participants from Al-Quds University (Al-Azhar branch) and 197 from The Islamic University of Gaza). During the 2019-2020 academic year, there were 818 basic-year medical students at the two universities, 418 at Al-Quds University Al-Azhar branch, Gaza, and 400 at The Islamic University of Gaza. Sample size was calculated using online calculator (calculator.net)^1^ in order to set the size of the sample needed. With 95% of confidence interval, 5% margin error and 50% expected frequency, the sample size needed from Al-Quds University (Al-Azhar branch) was 201 out of 418 and from The Islamic University of Gaza was 197 out of 400, which is the exact sample size of the research done.

We targeted male and female students in the basic years at both universities. Basic medicine involves the study of the basic structure and functions of the human body but not clinical medicine. Therefore, clinical-year students were excluded.

A non-probability accidental sampling technique was used. As a result of the COVID-19 pandemic, cities were placed under almost continuous curfew with the closure of all schools and universities. Communication with students shifted from direct communication to online methods, making access to students more difficult. Additionally, the Gaza Strip suffered particularly from power outages, and consequently internet outages, that forced us to choose a method that suited students and was within their abilities, meaning the accidental sampling technique was most suitable.

### Ethical procedure

Ethical and administrative approvals were obtained from the Dean of Scientific Research and Libraries at Al-Azhar University, Gaza, and the Ethical Committee at The Islamic University of Gaza. Consent forms were sent to the participants to obtain their agreement to participate in the study. They were told that their participation was voluntary, and they could withdraw from the study at any point during the process. Also, their participation was anonymous and no one would have access to their data except the main researchers.

### Data collection

The questionnaires, in the form of a Google Form, were published on the Facebook group of each level in both universities. Before accessing the questionnaire section, the participants were informed that their participation was voluntary, told the study objectives and given information about the instruments used. They were informed that their data would remain anonymous and confidential. If they agreed to participate, they were directed to the second section containing the questionnaire. The contact information of each data collector was also provided for them to ask about anything which was unclear.

### Study instruments

*Demographic variables*. Gender, academic performance (The question was how you can evaluate your academic performance? It was clear for the students that we are asking about their academic performance that they have achieved during the previous year. Medical students are evaluated annually), level of study, parents’ education (none, school education, higher education), parents’ job (employed, unemployed).

*Medical Student Stressor Questionnaire* (*MSSQ*). Consisting of 40 items, grouped into six domains: (1) academic-related stressors (ARS); (2) intrapersonal- and interpersonal-related stressors (IRS); (3) teaching- and learning-related stressors (TLRS); (4) social-related stressors (SRS); (5) drive- and desire-related stressors (DRS) and (6) group activities-related stressors (GARS) [30]. The MSSQ has good psychometric properties and is a valid and reliable instrument that can be used to identify students’ stressors and measure the intensity of the stressors. The mean domain scores are as follows: 0.00-1.00 = mild; 1.01-2.00 = moderate; 2.01-3.00 = high and 3.01-4.00 = severe. The Cronbach’s alpha is .918.

*Trait Emotional Intelligence Questionnaire—Short Form* (*TEIQue-SF*). Comprising 30 short statements designed to measure global trait emotional intelligence (trait EI), for example, “I’m a very motivated person”, “I change my mind often” or “I’m comfortable with the way I look” [31]. The original version of the questionnaire scoring is based on a 7-point Likert-type scale from 1 (strongly disagree) to 7 (strongly agree). The scoring scale is modified to a 5-point Likert-type scale. The participants put a mark (×) in the box that most accurately reflects their feelings: strongly agree = 4, agree = 3, neither agree nor disagree = 2, disagree = 1 and strongly disagree = 0. The Cronbach’s alpha is .792.

*The anxiety symptoms scale*. A brief measure of generalised anxiety disorder (GAD) [32]. A 7-item instrument score is calculated by assigning scores of 0, 1, 2 or 3 to the response categories “not at all”, “several days”, “more than half the days”, and “nearly every day”, then adding the scores. Scores of 5, 10 and 15 represent the cut-off points for mild, moderate and severe anxiety. When this is used as a screening tool, further evaluation is recommended if the score is 10 or greater. The Cronbach’s alpha is .860.

*Patient Health Questionnaire* (*PHQ-9 scale*). A self-administered version of the PRIME-MD diagnostic instrument for common mental disorders [33]. The PHQ-9 is a depression module which scores each of the 9 DSM-IV criteria from 0 (not at all) to 3 (nearly every day). The more symptoms the participants reported, the more severity the depression will be. The Cronbach’s alpha is .845.

### Statistical analysis

The data collected was entered into and then analysed using IBM Statistical Package for the Social Sciences (SPSS) version 25. *T* tests were used to investigate the differences between variables (e.g. male and female); one-way ANOVA was used to examine the differences amongst three or more variables (e.g. type of residence: city, camp or village). Pearson correlation coefficient analyses were performed to examine the association amongst the continuous variables (e.g. exposure to stressful life events and anxiety). Linear regression analyses were employed to examine the associations between mental health problems (anxiety and depression) as dependent variables (outcome) and demographic variables (e.g. age, gender) and exposure to stressful life events as independent (predictor) variables.

## Results

### Demographic data reporting

Of the targeted population, 379 medical students completed the questionnaire, of these, 57.5% were female, 38.8% from the first year, 29.8% from the second year and 31.4% from the third year. The majority of students (63.1%) evaluated their academic progress as “very good”, 21.9% evaluated it as “excellent”, 14.0% evaluated it as “good”, and 1.1% evaluated it as “acceptable”. Regarding parents’ education, only 0.8% of fathers and 1.1% of mothers were illiterate, 13.1% of fathers and 30.1% of mothers completed their school education and 85.8% of fathers and 68.9% of mothers finished their higher education. The majority of fathers work (81.3%), whereas 44.3% of mothers work (see Table 1).

**Table 1:** Frequency of demographic variables

	***N***	%
**Gender**		
Male	160	42.4
Female	218	57.5
**University name**		
Al-Azhar University	214	56.5
Islamic University	165	43.5
**Academic year**		
The first year	147	38.8
Second year	113	29.8
Third year	119	31.4
**Academic performance**		
Excellent	83	21.9
Very good	239	63.1
Good	53	14
Accepted	4	1.1
**Father education**		
None	3	0.8
School education	51	13.1
Higher education	325	85.8
**Father job**		
Unemployed	71	18.7
Employed	308	81.3
**Mother education**		
None	4	1.1
School education	114	30.1
Higher education	261	68.9
**Mother job**		
Unemployed	211	55.7
Employed	168	44.3

### Frequency of exposure to stress categories

The results show that the majority of students reported high to severe level of ARS. However, most reported mild to moderate DRS (see Table 2).

**Table 2:** Frequency of exposure to stress categories

Stress categories	Mild	Moderate	High	Severe
	***N*** (%)	***N*** (%)	***N*** (%)	***N*** (%)
ARS	-	44 (11.6)	245 (64.6)	90 (23.7)
IRS	31 (8.2)	118 (31.1)	171 (45.1)	59 (15.6)
TLRS	1 (0.3)	89 (23.5)	217 (57.3)	72 (19)
SRS	7 (1.8)	150 (39.6)	203 (53.6)	19 (5)
DRS	90 (23.7)	150 (39.6)	100 (26.4)	39 (10.3)
GARS	34 (9)	175 (46.2)	152 (40.1)	18 (4.7)

*ARS* academic-related stressors, *IRS* intrapersonal- and interpersonal-related stressors, *TLRS* teaching- and learning-related stressors, *SRS* social-related stressors, *DRS* drive- and desire-related stressors, *GARS* group activities-related stressors

### Demographic variables and exposure to stress, trait EI, anxiety and depression

There is a significant effect of gender amongst students regarding their exposure to: ARS, *t*(301.935) = 2.564, *p* = .01; TLRS, *t*(376) = .886, *p* = .009; SRS, *t*(376) = .381, *p* = .008; GARS, *t*(376) = 1.295, *p* = .01 and anxiety, *t*(376) = 2.593, *p* = .01. Female students show higher levels of ARS, TLRS, SRS, GARS and anxiety symptoms than male students. In contrast, the results reveal no significant differences by gender regarding IRS, DRS, trait ET or depression (see Table 3).

**Table 3:** Means and standards deviations according to gender

Stress categories	Male		Female		*P*
	*M*	*SD*	*M*	*SD*	
ARS	33.50	7.40	35.34	6.10	.009
IRS	15.86	5.64	15.83	5.29	.95
TLRS	16.90	4.31	18.05	4.21	.009
SRS	12.58	3.29	13.52	3.45	.008
DRS	5.65	2.91	5.55	2.79	.76
GARS	7.68	2.69	8.38	2.54	.01
Stress (total)	92.18	21.18	96.70	18.82	.02
Trait EI	95.88	11.72	95.22	12.20	.59
Anxiety	7.16	4.55	8.38	4.53	.01
Depression	10.01	5.23	10.67	5.74	.25

The results also reveal no significant differences in parents’ job regarding exposure to ARS, TLRS, SRS, GARS, IRS, DRS, trait ET, anxiety or depression.

The one-way ANOVA indicates a significant effect of academic year on trait EI, *F*(2, 376) = 3.35, *p* = .03. Least significant difference (LSD) post hoc tests reveal that first year medical students reported higher levels of trait emotional intelligence than third year students.

Academic performance is found to have a significant effect on the following: ARS, *F*(3,375) = 4.38, *p* = .005; DRS, *F*(3,375) = 11.33, *p* < .001; trait EI, *F*(3,375) = 8.22, *p* < .001; anxiety symptoms, *F*(3,375) = 4.06, *p* = .007 and depression symptoms, *F*(3,375) = 5.81, *p* = .001. LSD post hoc tests show that students with excellent academic performance reported lower levels of ARS than those with good academic performance (*p* = .001). Similarly, those with very good academic performance reported lower levels of ARS than those with good academic performance (*p* = .007). Students with excellent academic performance reported lower levels of DRS than those with very good academic performance (*p* = .003) and those with good academic performance (*p* < .001). Similarly, those with very good academic performance reported lower levels of DRS than those with good academic performance (*p* < .001).

Students with excellent academic performance reported higher levels of trait EI than those with good academic performance (*p* = .001) and those with acceptable academic performance (*p* < .001). Similarly, those with very good academic performance reported higher levels of trait EI than those with good academic performance (*p* = .004) and those with acceptable academic performance (*p* < .001). Likewise, those with good academic performance reported higher levels of trait EI than those with acceptable academic performance (*p* = .009).

Students with excellent academic performance reported lower levels of anxiety symptoms than those with good academic performance (*p* = .004). Similarly, those with very good academic performance reported lower levels of anxiety symptoms than those with good academic performance (*p* = .003). Students with excellent academic performance reported lower levels of depression symptoms than those with good academic performance (*p* < .001) and those with acceptable academic performance (*p* = .04). Similarly, those with very good academic performance reported lower levels of depression symptoms than those with good academic performance (*p* = .001). Those with good academic performance reported higher levels of trait EI than those with acceptable academic performance (*p* = .009).

Pearson’s correlation analyses were performed to investigate the association between several continuous demographic variables (academic year, academic performance), stress domains (ARS, IRS, TLRS, SRS, DRS, GARS), trait EI, anxiety and depression symptoms. The results indicate that academic year is negatively associated with academic performance, trait EI and TLRS. Students in higher academic years show lower levels of academic performance, trait EI and TLRS. Academic performance level is correlated positively with trait EI, and correlated negatively with stress domains (except IRS), total stress, anxiety symptoms and depression symptoms. Trait EI has a significant negative association with anxiety symptoms, depression symptoms, stress domains (ARS, IRS, TLRS, SRS, DRS, GARS) and total stress. Finally, stress domains are positively correlated with anxiety and depression symptoms (see Table 4).

**Table 4:** Correlation between stress domains, trait EI, anxiety and depression symptoms

	1	2	3	4	5	6	7	8	9	10	11	12
1. Academic year	-											
2. Academic performance	−.157**	-										
3. ARS	−.034	−.176**	-									
4. IRS	.005	−.085	.572**	-								
5. TLRS	−.102*	−.121*	.745**	.541**	-							
6. SRS	−.080	−.128*	.665**	.496**	.603**	-						
7. DRS	.048	−.275**	.436**	.358**	.393**	.350**	-					
8. GARS	.013	−.119*	.595**	.427**	.510**	.407**	.397**	-				
9. Stress (total)	−.037	−.185**	.907**	.773**	.839**	.763**	.583**	.684**	-			
10. Trait EI	−.131*	.212**	−.272**	−.184**	−.229**	−.202**	−310**	−410**	−.324**	-		
11. Anxiety	.067	−.147**	.322**	.254**	.248**	.217**	.262**	.286**	.343**	−.424**	-	
12. Depression	.017	−.186**	.355**	.227**	.360**	.200**	.324**	.312**	.380**	−.506**	.680**	-

**Correlation is significant at the 0.01 level (2-tailed)

*Correlation is significant at the 0.05 level (2-tailed)

### Prediction of anxiety and depression symptoms

Univariate and multivariate linear regression models were conducted to find the predictors of anxiety by exposure to stress. At first, the variables were entered individually into a series of simple univariate linear regression models (see Table 5). The variables showing significant prediction of anxiety were as follows: gender (female), *F*(1, 376) = 6.724, *p* = .01; academic level, *F*(1, 377) = 8.365, *p* = .004; academic-related stressors, *F*(1, 377) = 43.641, *p* < .001; intrapersonal- and interpersonal-related stressors, *F*(1, 377) = 26.051, *p* < .001; teaching- and learning-related stressors, *F*(1, 377) = 24.674, *p* < .001; social-related stressors, *F*(1, 377) = 18.549, *p* < .001; drive- and desire-related stressors, *F*(1, 377) = 27.768, *p* < .001; group activities-related stressors, *F*(1, 377) = 33.489, *p* < .001; stress (total), *F*(1, 377) = 50.236, *p* < .001 and trait emotional intelligence, *F*(1, 377) = 82.733, *p* < .001. A multiple regression model was created with the variables entered together. The overall model is significant, *F*(10, 367) = 12.509, *p* < .001, indicating that being female (*p* = .02), having a high level of academic-related stressors (*p* = .02) and having a low level of trait EI (*p* < .001) significantly predict a high level of anxiety symptoms (see Table 5).

**Table 5:** Hierarchical regression analysis for predicting anxiety and depression symptoms by exposure to stress (*N* = 378)

	Anxiety symptoms		Depression symptoms	
	Univariate	Multivariate	Univariate	Multivariate
	*B* [95% CI]	*B* [95% CI]	*B* [95% CI]	*B* [95% CI]
Gender	1.22 [.297, 2.15]*	1.04 [.198, 1.88]*	.656 [−.477, 1.78]	.278 [−.679, 1.23]
Study level	.368 [−.185, .922]	.133 [−.389, .615]	.115 [−.555, 0.785]	−.224 [−.794, 0.345]
Academic level	−1.06 [−1.79, −.342]*	−.162 [−.846, .523]	−1.63 [−2.05, −.760]***	−.390 [−1.16, .386]
ARS	.219 [.154, .284]***	.123 [.015, .231]*	.291 [.213, .369]***	.114 [−.021, .249]
IRS	.214 [.132, .297]***	.090 [−.006, .186]	.231 [ .131, .332]***	.002 [−.107, .111]
TLRS	.264 [.160, .369]***	−.032 [−.182, .117]	.463 [.342, .585]***	.268 [.084, .459]**
SRS	.290 [.158, .422]***	−.047 [−.213, .118]	.323 [.162, .483]***	−.203 [−.410, .005]
DRS	.422 [.265, .580]***	.101 [−.069, .272]	.632 [.446, .819]***	.320 [.109, .531]**
GARS	.496 [.327, .664]***	−.032 [−.240, .175]	.654 [.452, .855]***	−.106 [−.341, 0.129]
Stress (total)	.079 [.057, .100]***		.105 [.079, .131]***	
Trait EI	−.161 [−.196, −.126]***	−.135 [−.174, −.097]***	−.232 [−.272, −.192]***	−.198 [−.241, −.155]***
*R*^*2*^		.254		0.586
Adjusted *R*^*2*^		.234		0.343

***p < .001

**p < .01

*p < .05

Univariate and multivariate linear regression models were also conducted to find the predictors of depression symptoms by exposure to stress. The variables were entered individually into a series of simple univariate linear regression models (see Table 5). The variables showing significant prediction of depression in univariate regression are as follows: academic level, *F*(1, 377) = 13.549, *p* < .001; academic-related stressors, *F*(1, 377) = 54.205, *p* < .001; intrapersonal- and interpersonal-related stressors, *F*(1, 377) = 20.555, *p* < .001; teaching- and learning-related stressors, *F*(1, 377) = 56.020, *p* < .001; social-related stressors, *F*(1, 377) = 15.627, *p* < .001; drive- and desire-related stressors, *F*(1, 377) = 44.347, *p* < .001; group activities-related stressors, *F*(1, 377) = 40.514, *p* < .001; stress (total), *F*(1, 377) = 63.603, *p* < .001 and trait emotional intelligence, *F*(1, 377) = 129.531, *p* < .001. A multiple regression model was created with the variables entered together. The overall model is significant, *F*(10, 367) = 19.191, *p* < .001, indicating that having a high level of teaching and learning-related stressors (*p* < .002), social-related stressors (*p* < .02) and drive- and desire-related stressors (*p* < .04) and a low level of trait EI (*p* < .001), significantly predict high level of depression symptoms (see Table 5).

## Discussion

The aim of the current study is to investigate the relationship between exposure to stress, trait EI and mental health (depression and anxiety symptoms) amongst basic-year medical students during the COVID-19 pandemic in the Gaza Strip. To the best of our knowledge, this is the first study assessing exposure to stress amongst basic-year medical students during the COVID-19 pandemic in the Gaza Strip. Previous research shows that medical students may be exposed to stressful events during their studies and they experience a significant level of stress in United Arab Emirates, Egypt, Kingdom of Saudi Arabia [7] and Malaysia [8]. However, levels of stress and their consequences have not previously been investigated in the Gaza Strip, especially not during the COVID-19 pandemic which may increase stressors for medical students and consequently increase anxiety and depression symptoms.

The results show that the majority of students reported high to severe academic-related stressor and teaching- and learning-related stressor, moderate to high interpersonal- and intrapersonal-related stressor, social-related stressor and group activities-related stressor. Meanwhile, drive- and desire-related stressor was the least reported by students. These findings could be attributed to the fact that medical colleges in Gaza accept only students with excellent academic performance, who inevitably try their best to keep their performance at an excellent level. In addition to experiencing a new academic life, students in this period were also exposed to academic and teaching stressors such as shifting to e-learning, faced with weak internet networks, power outages and low levels of access to the internet. All these stressors mean students reported high to severe levels of academic-related stressor and teaching- and learning-related stressor.

Female students reported higher levels of academic-related stressor, teaching- and learning-related stressor, social-related stressor, group activities-related stressor and anxiety symptoms than male students. The results are in the line with previous studies which show that female medical students report higher levels of stress than male medical students [34, 35]. In contrast, the results reveal no significant differences in gender regarding interpersonal- and intrapersonal-related stressor, drive- and desire-related stressor, trait ET or depression.

The results show that students with higher levels of academic performance show lower levels of academic-related stressor, drive- and desire-related stressor, anxiety and depression symptoms. This means that exposure to academic-related stressor and drive- and desire-related stressor may have a negative effect on student performance. Hence, students may get lower grades and consequently show higher levels of anxiety and depression symptoms. This is consistent with previous studies which show a correlation between exposure to high levels of stressors and low-grade point averages [12]. Higher levels of academic performance appear to be consistent with higher levels of trait EI and lower levels of academic-related stressor and drive- and desire-related stressor compared to those with lower levels of trait EI. In other words, students with higher levels of trait EI can better manage exposure to stress. Accordingly, these students can achieve high levels of academic performance and hence show lower levels of anxiety and depression symptoms. These findings are consistent with previous research which shows that higher levels of trait EI decrease the effects of exposure to stress [36], more likely to show persistence, which decreases the likelihood of school burnout [37], report lower degrees of burnout and somatic complaints when dealing with emotional labour [38]. Finally, we conclude with a study which finds a positive relation between trait EI and career adaptability, as well as academic participation [39].

Transition from year one to the following years is negatively associated with level of academic performance and trait EI, probably due to difficulties arising in academic life with each year, meaning higher academic-related stressor. On the other hand, teaching- and learning-related stressor is negatively associated with transition from year one to the following years, which could be related to students getting used to the teaching system in the university.

These results are in-line with previous studies (e.g. [40]) which indicate that more exposure to stressors lead to lower academic performance for medical students. During the COVID-19 pandemic, the universities in Gaza moved to online-only teaching via Google Meet and Moodle. This shift came at a time when Gaza faced several obstacles to online learning, including weak internet networks, power outages, insufficient awareness amongst students and their families of the importance of online learning and low levels of access to online material and computers or smartphones for some [27]. As a result, students with higher levels of exposure to stress report higher levels of anxiety and depression symptoms.

The results of the regression analysis show that exposure to stress significantly predicts mental health problem, specifically anxiety symptoms and depression symptoms [6, 15]. Continuous exposure to stress is found to be a risk factor which increases the tendency for anxiety and depression symptoms [7, 10]. However, when we adjust all the variables together, exposure to academic-related stressor is a significant predictor of anxiety symptoms, whilst exposure to teaching- and learning-related stressor and group activities-related stressor are significant predictors of depression symptoms.

## Conclusions

To the best of our knowledge, this is the first study assessing exposure to stress amongst basic-year medical students during the COVID-19 pandemic in the Gaza Strip. To conclude, basic-year medical students in the Gaza Strip are exposed to stressful events which cause higher levels of anxiety and depression symptoms. This is important for policy makers in medical colleges in Palestinian universities, who should take into account the stressors that basic-year medical students are exposed to during their studies and provide counselling programmes to help them manage these stressors. Furthermore, medical schools and health authorities should offer early detection and prevention programmes and interventions for depression for medical students before graduation [41].

## Limitations and strengths of the study

The current study has some limitations. Firstly, it is a cross-sectional study, and, therefore, no causal relationships can be drawn [42]. Future studies could address this by applying longitudinal designs. Secondly, the data was collected by self-reported questionnaire, and thus, there is the potential for reporting bias. Thirdly, the study was undertaken on students in two universities in the Gaza Strip, so the results may not be generalisable to other universities in Palestine. However, to establish a true baseline for Palestinian medical schools, this research should have been conducted at a national level in all Palestinian medical schools. Finally, depressive and anxiety symptoms are assessed on self-administered scales. Future studies could apply clinical assessments to assess anxiety and depression amongst medical college students.

## Data Availability

The datasets used and/or analysed during the current study are available from the corresponding author on reasonable request.

## Abbreviations

Trait EI: Trait emotional intelligence
ARS: Academic-related stressors
IRS: Intrapersonal- and interpersonal-related stressors
TLRS: Teaching- and learning-related stressors
SRS: Social-related stressors
DRS: Drive- and desire-related stressors
GARS: Group activities-related stressors

## Footnotes

1 https://www.calculator.net/

## References

[1] Zeng W, Chen R, Wang X, Zhang Q, Deng W (2019). Prevalence of mental health problems among medical students in China: a meta-analysis. Medicine (Baltimore).

[2] Nechita F, Nechita D, Pîrlog MC, Rogoveanu I (2014). Stress in medical students. Romanian J Morphol Embryol Rev Roum Morphol Embryol.

[3] Zegeye A, Mossie A, Gebrie A, Markos Y (2018) Stress among postgraduate students and its association with substance use. J Psychiatry 21(3). 10.4172/2378-5756.1000448

[4] Krohne HW (2001). Stress and Coping Theories. Stress and coping theories.

[5] Azza Y, Grueschow M, Karlen W, Seifritz E, Kleim B (2020) How stress affects sleep and mental health: nocturnal heart rate increases during prolonged stress and interacts with childhood trauma exposure to predict anxiety. Sleep 43:zsz310. 43(6). 10.1093/sleep/zsz310 10.1093/sleep/zsz310 31863098

[6] Tian-Ci Quek T, Wai-San Tam W, X Tran B, et al (2019) The global prevalence of anxiety among medical students: a meta-analysis. Int J Environ Res Public Health 16. 10.3390/ijerph16152735, 15 10.3390/ijerph16152735 PMC6696211 31370266

[7] Sehlo M, Al-Zaben F, Khalifa D et al (2018) Stress among medical students in a college of medicine in Saudi Arabia: sex differences. Middle East Curr Psychiatry 25(4):150–154. 10.1097/01.XME.0000542433.59065.97

[8] Sherina MS (2004). Psychological stress among undergraduate medical students..

[9] Pagnin D, de Queiroz V (2015) Comparison of quality of life between medical students and young general populations. Educ Health 28(3):209–212. 10.4103/1357-6283.178599 10.4103/1357-6283.178599 26996647

[10] Wang C, Zhao H (2020) The impact of COVID-19 on anxiety in Chinese university students. Front Psychol 11. 11. 10.3389/fpsyg.2020.01168 10.3389/fpsyg.2020.01168 PMC7259378 32574244

[11] Alharbi H, Almalki A, Alabdan F, Haddad B (2018) Depression among medical students in Saudi medical colleges: a cross-sectional study. Adv Med Educ Pract 9:887–891. 10.2147/AMEP.S182960 10.2147/AMEP.S182960 PMC6287521 30584384

[12] Shadid A, Shadid AM, Shadid A, Almutairi FE, Almotairi KE, Aldarwish T, Alzamil O, Alkholaiwi F, Khan SUD (2020) Stress, burnout, and associated risk factors in medical students. Cureus 12:e6633. 10.7759/cureus.6633 10.7759/cureus.6633 PMC6957047 31966946

[13] Dyrbye LN, Thomas MR, Shanafelt TD (2006) Systematic review of depression, anxiety, and other indicators of psychological distress among U.S. and Canadian medical students. Acad Med J Assoc Am Med Coll 81(4):354–373. 10.1097/00001888-200604000-00009 10.1097/00001888-200604000-00009 16565188

[14] Quek TTC, Tam WWS, Tran BX, Zhang M, Zhang Z, Ho CS, Ho RC (2019) The global prevalence of anxiety among medical students: a meta-analysis. Int J Environ Res Public Health 16(15). 10.3390/ijerph16152735 10.3390/ijerph16152735 PMC6696211 31370266

[15] Shawahna R, Hattab S, Al-Shafei R, Tab’ouni M (2020) Prevalence and factors associated with depressive and anxiety symptoms among Palestinian medical students. BMC Psychiatry 20(1):244. 10.1186/s12888-020-02658-1 10.1186/s12888-020-02658-1 PMC7236464 32429889

[16] Alswerki MN, Alwali A, Hamouda M, et al (2018) Prevalence of stress, anxiety, and depression among medical students in Gaza Strip: a cross-sectional descriptive study. 10.13140/RG.2.2.17484.46723

[17] Petrides K, Pita R (1953) Kokkinaki F (2007) The location of trait emotional intelligence in personality factor space. Br J Psychol Lond Engl 98(2):273–289. 10.1348/000712606X120618 10.1348/000712606X120618 17456273

[18] Sánchez-Ruiz MJ, Hernández-Torrano D, Pérez-González JC, Batey M, Petrides KV (2011). The relationship between trait emotional intelligence and creativity across subject domains. Motiv Emot.

[19] López-Pina JA, Hernández D, Sáinz M, et al (2010) Trait emotional intelligence and academic performance: controlling for the effects of

[20] Fiorilli C, Farina E, Buonomo I, Costa S, Romano L, Larcan R, Petrides KV (2020). Trait emotional intelligence and school burnout: the mediating role of resilience and academic anxiety in high school. Int J Environ Res Public Health.

[21] Poulou MS (2014). How are trait emotional intelligence and social skills related to emotional and behavioural difficulties in adolescents?. Educ Psychol.

[22] Bright JEH, Pryor RGL (2011). The chaos theory of careers. J Employ Couns.

[23] Nikooyeh E, Zarani F, Fathabadi J (2017). The mediating role of social skills and sensation seeking in the relationship between trait emotional intelligence and school adjustment in adolescents. J Adolesc.

[24] Frederickson N, Petrides KV, Simmonds E (2012). Trait emotional intelligence as a predictor of socioemotional outcomes in early adolescence. Personal Individ Differ.

[25] Malouff JM, Schutte NS, Thorsteinsson EB (2014). Trait emotional intelligence and romantic relationship satisfaction: a meta-analysis. Am J Fam Ther.

[26] OCHA (2021). COVID-19 emergency situation report 28 | February 2021.

[27] UNESCO (2020). COVID-19 in Palestine: how distance learning will help student continue education.

[28] Palestinian Central Bureau of Statistics (2017) Palestine in figures 2016

[29] Ministry of Health (2021) Daily report. http://www.moh.gov.ps/portal.

[30] Yusoff MSB, Abdul Rahim AF, Yaacob MJ (2010). Prevalence and sources of stress among Universiti Sains Malaysia medical students. Malays J Med Sci MJMS.

[31] Petrides KV, Sangareau Y, Furnham A, Frederickson N (2006). Trait emotional intelligence and children’s peer relations at school. Soc Dev.

[32] Spitzer RL, Kroenke K, Williams JBW, Löwe B (2006) A brief measure for assessing generalized anxiety disorder: the GAD-7. Arch Intern Med 166(10):1092–1097. 10.1001/archinte.166.10.1092 10.1001/archinte.166.10.1092 16717171

[33] Kroenke K, Spitzer RL, Williams JB (2001) The PHQ-9: validity of a brief depression severity measure. J Gen Intern Med 16(9):606–613. 10.1046/j.1525-1497.2001.016009606.x 10.1046/j.1525-1497.2001.016009606.x PMC1495268 11556941

[34] Rosiek A, Rosiek-Kryszewska A, Leksowski Ł, Leksowski K (2016) Chronic stress and suicidal thinking among medical students. Int J Environ Res Public Health 13(2):212. 10.3390/ijerph13020212 10.3390/ijerph13020212 PMC4772232 26891311

[35] Shah M, Hasan S, Malik S, Sreeramareddy CT (2010) Perceived stress, sources and severity of stress among medical undergraduates in a Pakistani medical school. BMC Med Educ 10(1):2. 10.1186/1472-6920-10-2 10.1186/1472-6920-10-2 PMC2820489 20078853

[36] Ranasinghe P, Wathurapatha WS, Mathangasinghe Y, Ponnamperuma G (2017) Emotional intelligence, perceived stress and academic performance of Sri Lankan medical undergraduates. BMC Med Educ 17(1):41. 10.1186/s12909-017-0884-5 10.1186/s12909-017-0884-5 PMC5319135 28219419

[37] Sanchez-Ruiz M-J, Baaklini A (2018). Individual and social correlates of aggressive behavior in Lebanese undergraduates: the role of trait emotional intelligence. J Soc Psychol.

[38] Mikolajczak M, Menil C, Luminet O (2007). Explaining the protective effect of trait emotional intelligence regarding occupational stress: exploration of emotional labour processes. J Res Personal.

[39] Merino-Tejedor E, Hontangas PM, Petrides KV (2018). La adaptabilidad a la carrera media el efecto de la inteligencia emocional rasgo sobre el compromiso académico. Rev Psicodidáct.

[40] Crego A, Carrillo-Diaz M, Armfield JM, Romero M (2016) Stress and academic performance in dental students: the role of coping strategies and examination-related self-efficacy. J Dent Educ 80(2):165–172. 10.1002/j.0022-0337.2016.80.2.tb06072.x 26834134

[41] Puthran R, Zhang MWB, Tam WW, Ho RC (2016) Prevalence of depression amongst medical students: a meta-analysis. Med Educ 50(4):456–468. 10.1111/medu.12962 10.1111/medu.12962 26995484

[42] Baron RM, Kenny DA (1986) The moderator–mediator variable distinction in social psychological research: conceptual, strategic, and statistical considerations. J Pers Soc Psychol 51(6):1173–1182. 10.1037/0022-3514.51.6.1173 10.1037//0022-3514.51.6.1173 3806354

